# A Meta-analysis of the effects of Exercise Training on Left Ventricular Remodeling Following Myocardial Infarction: Start early and go longer for greatest exercise benefits on remodeling

**DOI:** 10.1186/1745-6215-12-92

**Published:** 2011-04-04

**Authors:** Mark Haykowsky, Jessica Scott, Ben Esch, Don Schopflocher, Jonathan Myers, Ian Paterson, Darren Warburton, Lee Jones, Alexander M Clark

**Affiliations:** 1 Faculty of Rehabilitation Medicine, University of Alberta, Edmonton, Canada; 2 Cardiovascular Physiology and Rehabilitation Laboratory, University of British Columbia, Vancouver, British Columbia, Canada; 3 School of Public Health Sciences, University of Alberta, Edmonton, AB, Canada; 4 Cardiology Division (111C), VA Palo Alto Health Care System, Stanford University, Palo Alto, CA, USA; 5 Faculty of Medicine, University of Alberta, Edmonton, AB, Canada; 6 Department of Radiation Oncology, Duke University Medical Center, Durham, NC, USA; 7 Faculty of Nursing, University of Alberta, Edmonton, AB, Canada

## Abstract

**Background:**

The effects of variations in exercise training on Left ventricular (LV) remodeling in patients shortly after Myocardial Infarction (MI) are important but poorly understood.

**Methods:**

Systematic review incorporating meta-analysis using meta-regression. Studies were identified via systematic searches of: OVID MEDLINE (1950 to 2009), Cochrane Central Register of Controlled Trials (1991 to 2009), AMED (1985 to 2009), EMBASE (1988 to 2009), PUBMED (1966 to 2009), SPORT DISCUS (1975 to 2009), SCOPUS (1950 to 2009) and WEB OF SCIENCE (1950 to 2009) using the medical subject headings: myocardial infarction, post myocardial infarction, post infarction, heart attack, ventricular remodeling, ventricular volumes, ejection fraction, left ventricular function, exercise, exercise therapy, kinesiotherapy, exercise training. Reference lists of all identified studies were also manually searched for further relevant studies. Studies selected were randomized controlled trials of exercise training interventions reporting ejection fraction (EF) and/or ventricular volumes in patients following recent MI (≤ 3 months) post-MI patients involving control groups. Studies were excluded if they were not randomized, did not have a 'usual-care' control (involving no exercise), evaluated a non-exercise intervention, or did not involve human subjects. Non-English studies were also excluded.

**Results:**

After screening of 1029 trials, trials were identified that reported EF (12 trials, n = 647), End Systolic Volumes (ESV) (9 trials, n = 475) and End Diastolic Volumes (EDV) (10 trials, n = 512). Meta-regression identified that changes in EF effect size difference decreased as the time between MI and initiation of the exercise program lengthened, and increased as the duration of the program increased (Q = 25.48, df = 2, p < 0.01, R^2 ^= 0.76). Greater reductions in ESV and EDV (as indicated by effect size decreases) occurred with earlier initiation of exercise training and with longer training durations (ESV: Q = 23.89, df = 2, p < 0.05, R^2 ^= 0.79; EDV: Q = 27.42, df = 2, p < 0.01, R^2 ^= 0.83). Differences remained following sensitivity analysis. Each week that exercise was delayed required an additional month of training to achieve the same level of benefit on LV remodeling.

**Conclusions:**

Exercise training has beneficial effects on LV remodeling in clinically stable post-MI patients with greatest benefits occurring when training starts earlier following MI (from one week) and lasts longer than 3 months.

## Background

There is strong and consistent evidence that exercise after a myocardial infarction (MI) improves overall and cardiovascular-related mortality [1,2]. In low, middle and high income countries, patients who exercise regularly are over 30% less likely to experience further MI, stroke or death [3]. However, the mechanisms of these mortality benefits are not well understood.

Left ventricular (LV) remodeling is an accurate predictor of cardiac mortality following MI [4] but it is not clear how exercise effects LV remodeling. In some past trials, aerobic training led to decreases in LV end-diastolic (EDV) and end-systolic volumes (ESV) and increases in ejection fraction (EF) [5-7]. In other trials, exercise training led to increased LV volumes [8,9] and decreased EF [8]. Still further, other trials identified that following MI exercise training does not alter LV volumes [10-14] or EF [11-14]. Though the effects of LV remodeling are likely to vary based on the characteristics of populations and exercise interventions, it is not known why these variations occur [15].

Understanding these inconsistencies and the effects of exercise on LV remodeling is important because this knowledge could be used to heighten the benefits of exercise after MI. Though arguably essential for effective support and health services, recent guidelines on physical activity after MI do not provide any recommendations on the key but basic issues of when exercise should commence after MI and how long supervised exercise should continue to ensure or maximize benefits [2]. Information on these design characteristics should provide more effective and useful evidence for health professionals [16]. Consequently we performed a systematic review and meta-analysis to assess the overall effects of exercise training on LV remodeling in clinically stable post-MI patients.

## Methods

### Data sources

A searched was undertaken of OVID MEDLINE (1950 to 2009), Cochrane Central Register of Controlled Trials (1991 to 2009), AMED (1985 to 2009), EMBASE (1988 to 2009), PUBMED (1966 to 2009), SPORT DISCUS (1975 to 2009), SCOPUS (1950 to 2009) and WEB OF SCIENCE (1950 to 2009) using the following medical subject headings: myocardial infarction, post myocardial infarction, post infarction, heart attack, ventricular remodeling, ventricular volumes, ejection fraction, left ventricular function, exercise, exercise therapy, kinesiotherapy, exercise training. Reference lists of all identified studies were also manually searched for further relevant investigations.

### Study selection

Two investigators (MH and AC) independently reviewed the titles and abstracts of all citations to identify studies reporting the effect of exercise training on EF and/or ventricular volumes in recent (≤ 3 months) post-MI patients. We excluded trials that were not randomized, did not have a usual care control group, non-exercise intervention, exercise and other intervention, and non-human studies. We also excluded non-English articles.

### Data extraction and quality assessment

Two authors (MH and BE) extracted relevant outcome data and any disagreement was resolved by consensus in discussion with AC. When necessary, original investigators were contacted to clarify data or provide additional data; authors for 3 studies provided further information. Quality was assessed using the Jadad scale [17].

### Data synthesis and analysis

All data was entered into SPSS files and analyzed with SPSS v15 software utilizing meta-analysis and meta-regression scripts created by Lipsey and Wilson [18]. The review conforms to the requirements of PRISMA reporting standards (Additional file 1). Formulae for calculation of outcomes is provided in Additional File 2.

## Results

### Study selection

**Figure 1:**
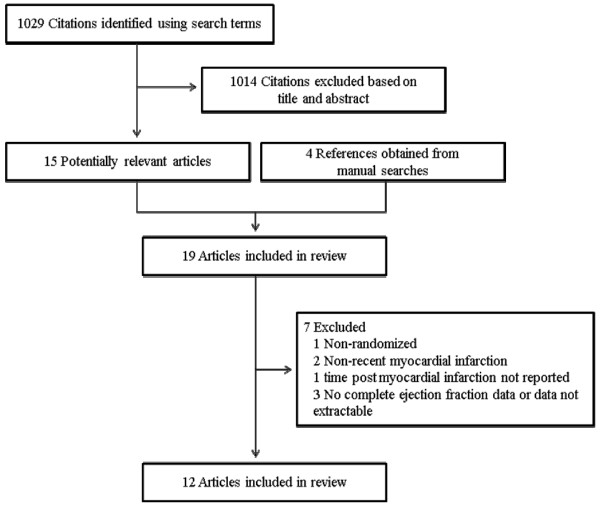
**Flow of trials through the selection process**.

After initial review of 1,033 citations (Figure 1), 19 papers were reviewed in full; of these, seven were excluded. Reasons for this exclusion were: EF data incomplete or un-extractable (n = 3), non-randomized design (n = 1), time from MI to initiation of exercise training was unreported (n = 1), inclusion of patients experiencing MI >3 months (n = 2). One trial [19] randomly assigned subjects 'a priori' to exercise or control groups based on baseline EF (≤ 30 and >30%). Accordingly, data for these sub-groups were analyzed separately.

### Study characteristics

The selected studies [5-7,9-14,19-21](Additional File 3) contained 647 post-MI patients (mean age: 55 years) with impaired LV systolic function (weighted mean EF = 44%). Trials incorporated aerobic training at 60% to 80% of baseline peak oxygen uptake (or heart rate) for 20 to 180 minutes per session (Table 1). The length of training program was 1 to 6 months in duration (Table 1). All trials reported pre and post measures for outcome variable for both treatment groups and for control groups. No trial described randomization procedures or blinding methods. Consequently, trials tended to be assessed as being of low to moderate quality.

**Table 1:** Description of exercise training program

Study	Frequency (days/week)	Intensity	Exercise duration (min/session)	Mode	Program length (mn)
Giallauria et al [6]	3	60-70% VO_2peak_	30	Cycle	6
Giallauria et al [5]	3	60-70% VO_2peak_	30	Cycle	6
Giallauria et al [12]	3	70% VO_2peak_	30	Cycle	3
Giallauria et al [13]	3	60% VO_2peak_	30	Cycle	3
Giannuzzi et al [7]	3-7	80% peak HR	30 >30 min	Cycle Walk	6
Koizumi et al [14]	7	Moderate speed	30	Walk	3
Kubo et al [9]	3	HR at VT	20 min, twice/day	Cycle Walk	3
Dubach et al [11]	4 - 7	60-70% HRR	45 120	Cycle Walk	2
Giannuzzi et al [10]	3-7	80% peak HR	30 >30	Cycle Walk	6
Heldal et al [20]	5	85% peak HR	120	Cycle Jogging	1
Jette et al [19]	7	70-80% peak HR	45-105	Cycle, Jogging Walk, Calisthenics	1
Grodzinski et al [21]	5	80% peak HR	30-180	Cycle, Jog Walk, Swim Calisthenics	1

(Heart rate; HRR: Heart rate reserve; VO_2peak_, peak oxygen consumption; VT: Ventilation threshold)

Three outcome variables were chosen for analysis. The primary variable was EF for which 12 trials reported data (n = 647). The secondary outcome variables were ESV and EDV for which data was only provided for a subset of trials: ESV (9 trials, n = 475) and EDV (10 trials, n = 512).

### Initial analysis

As the studies had high levels of statistical, clinical and methodological heterogeneity - as evident for example in the initial meta-analysis of EF (Cochran's Q = 33.57, df = 12, p < 0.01) and variations in effect size differences for each outcome variable, pooling of the results from the trials in a sub-analysis was inappropriate [22]. Therefore, we performed meta-regression of post-infarct LV remodeling. The effects of elements of the exercise interventions identified a priori for analysis were: time post-MI to initiation of exercise training program and length of training program [23].

### Exercise training and ejection fraction

**Figure 2:**
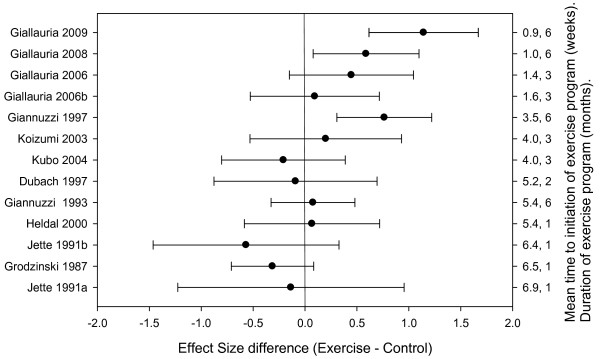
**EF effect size difference for individual trials categorized by time to exercise training and duration of training program**.

Meta-regression identified that change in EF effect size difference decreased as the time between MI and initiation of the exercise program lengthened, and increased as the duration of the program increased (Figure 2). Overall, these changes accounted for a significant proportion of the variations in outcomes across the studies (Q = 25.48, df = 2, p < 0.01, R^2 ^= 0.76) (Table 2). That is, when study level differences in average time to initiate and average length of program are considered the trials become comparable.

**Table 2:** Meta-Regression of the change in EF effect size difference

	B	Standard Error	Probability
Constant	0.314	0.315	ns
Time to Initiate	-0.119	0.045	p < 0.05
Length	0.098	0.046	p < 0.05

### Exercise training and ventricular volumes

**Figure 3:**
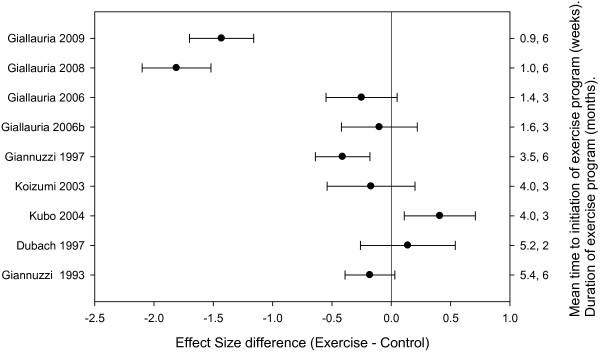
**ESV effect size difference for individual trials categorized by time to exercise training and duration of training program**.

**Figure 4:**
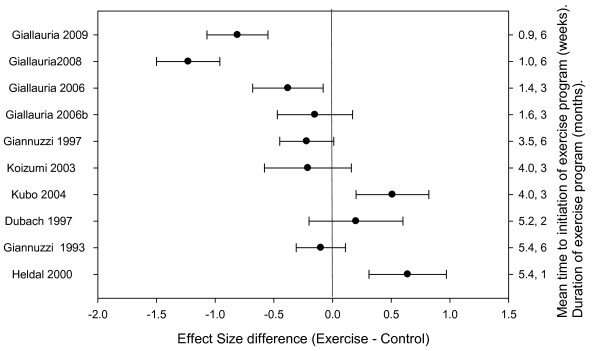
**EDV effect size difference for individual trials categorized by time to exercise training and duration of training program**.

A sufficient number of trials reported data on ESV (9 trials, n = 475) [5-7,9-14] and EDV (10 trials, n = 512) [5-7,9-14,20] to allow meta-regression of these outcomes. Similar to EF, effects on LV volumes were not homogeneous across studies (ESV, Q = 30.12, df = 8, p < 0.01; EDV, Q = 33.03, df = 9, p < 0.01). Greater reductions in ESV and EDV (as indicated by effect size decreases) occurred with earlier initiation of exercise training and with longer training durations (Tables 3 and 4, Figures 3 and 4). These differences accounted for a significant proportion of the heterogeneity for ESV (Q = 23.89, df = 2, p < 0.05, R^2 ^= 0.79) and EDV (Q = 27.42, df = 2, p < 0.01, R^2 ^= 0.83). As with EF effect size, when study level differences in average time to initiate and average length of program were considered, effects on ESV and EDV became comparable.

**Table 3:** Meta-Regression of the change in ESV effect size difference

	B	Standard Error	Probability
Constant	-0.07	0.45	ns
Time to Initiate	0.246	0.072	p < 0.01
Length	-0.249	0.078	p < 0.01

**Table 4:** Meta-Regression of the change in EDV effect size difference

	B	Standard Error	Probability
Constant	-0.186	0.54	ns
Time to Initiate	0.189	0.051	p < 0.01
Length	-0.148	0.051	p < 0.01

### Sensitivity Analysis

The findings may be influenced strongly by a small number of trials which evaluated exercise training after around one week. Analyses were therefore repeated excluding the four trials with the shortest latency to initiation of the exercise program [5,6,12,13]. After this removal, the analysis no longer had sufficient power to demonstrate significant effects. However, the regression coefficients remained of similar size and direction (For time to initiate: -0.119 to -0.178 (p < 0.15); for length from 0.098 to 0.069 (p < 0.25)) suggesting that the trends identified in the full analysis remained even when these trials are excluded. Removing the same trials from the analyses of ESV and EDV reduced the number of trials in the analyses more dramatically (5 and 6 trials respectively). In both re-analyses, the coefficients for time to initiate again remained in the same direction but decreased substantially in magnitude (ESV: 0.246 to 0.076 and EDV: 0.189 to 0.059) and were no longer significant. Yet, the coefficients for program length were more stable (ESV: -0.249 to -0.138, p < 0.10, and EDV: -0.148 to -0.13, p < 0.05). Hence, particularly for LV remodeling, the influence of time to initiation of exercise training after MI and subsequent length of training program remained after removal of the trials.

## Discussion

This systematic review found that exercise training had beneficial effects on LV remodeling after myocardial infarction but that the sizes of changes were dependent on time of instigation and duration of the exercise intervention. The largest changes in LV remodeling were obtained when programs began after around 1-week post MI hospital discharge and lasted for 6 months. For ESV, a strong predicator of mortality post-MI [4], each one-week delay in initiating exercise training would require an additional month of training to obtain a comparable reduction in ESV. Similarly, delaying exercise training by one week after a MI would require an additional month of training to attain the same change in LVEF.

As with all systematic reviews, the conclusions of this review are only as strong as the quality of the component studies. The trials included predominantly younger males with reduced systolic function though there is unlikely to be differences in effects by sex, future trials should include a greater proportion of women. In the trials, the type and location of infarction was not widely used to stratify results and few studies measured exercise capacity. Accordingly, future trials are needed to measure and assess the specific effects of these factors on LV remodeling. The trials included were not well described and did not report all of the information required to conduct optimal analysis in the case of repeated measures. Here, a conservative alternative nevertheless showed significant findings in the meta-regression. The effect sizes would have been larger if correlations between pre-and-post intervention scores had been available.

The findings are biologically plausible. Though the physiological mechanisms responsible for the anti-remodeling benefits of exercise training following MI are not well understood, they may arise from favorable improvements in coronary and peripheral vascular endothelial function, myocardial contractility, autonomic balance, or systolic and diastolic wall stress [24,25]. Several studies have also shown that aerobic training can elicit improvements in diastolic function and wall stress [5,12,13,26]. Post-training decreases in plasma pro-NT-BNP reduce EDV [5] and increase peak early mitral flow velocity [5,12,13] and peak early to late mitral flow velocity ratio [12,13]. Aerobic-training improves autonomic balance [27] and peripheral vascular endothelial function [28]. The anti-remodeling benefits arising from exercise programs with a longer training length may explain the effects of cardiac rehabilitation attendance on survival after MI [29].

### Clinical implications

There is no current recommendations [30] or consensus [31] as to when exercise training should commence after MI. Most cardiac rehabilitation and secondary prevention programs commence at least four to six weeks after hospital discharge [32]. To achieve maximal anti-remodeling benefits, clinically stable patients after uncomplicated MI should begin aerobic exercise training earlier after hospital discharge (from one week) and should continue training for up to 6 months. As this conclusion represents a potentially significant change from current practice, it is important to take into account the size and safety of this.

Though understandable, there is no evidence from the trials in this review or other observational studies that early commencement of exercise training is harmful. Trials have indicated that risk of adverse events or complications (including: re-infarction, and revascularization ) and EF are not raised by earlier physical activity when compared to activity after 6 weeks - even when pre-discharge (Bruce protocol) stress tests are performed 1-week post-MI [33]. Consistent with these findings, pre-discharge exercise (Bruce protocol) stress testing is safe and feasible in the majority of post-MI patients 3 days after infarction [34]. In trials in this review, no adverse events occurred during the 6-month exercise training sessions initiated at the earliest juncture (around one week post-MI) in people with mild to moderate LV systolic dysfunction [5,6]. Moreover, clinical events were significantly lower in the trained versus control group during the 6-month period.

In addition to benefitting mortality and being safe, earlier commencement of exercise training appears to markedly increase participation in secondary prevention services. Emerging evidence from observational studies indicates that commencement of cardiac rehabilitation after one week leads to a 90% increase in participation rates compared to a commencement after four weeks [35] and a faster return to work [33].

By providing more specific guidance on basic yet pivotal program design characteristics, these findings are also likely to render existing research evidence on exercise after MI more usable to health professionals and decision-makers [16]. Despite convincing evidence of the benefits of exercise after myocardial infarction [1,2,36,37], secondary prevention in clinical populations remains poor [38] and health services to promote exercise are under-utilized and poorly funded [39]. Health services, including different forms of cardiac rehabilitation [36,37,40] should be used more widely to promote exercise earlier and for longer after MI.

## Conclusions

Exercise training has beneficial effects on LV remodeling after myocardial infarction. Exercise earlier after infarction (around 1 week) and for longer improved LV remodeling. There is a need for future high quality randomized controlled trials of exercise intervention early post myocardial infarction on LV remodeling.

## Competing interests

The authors declare that they have no competing interests.

## Authors' contributions

Conception: MH, AMC, DS; Design: MH, AMC, DS; Acquisition of data: MH, AMC, DS, JS, BE.; Analysis:: MH, AMC, DS; Interpretation of data: MH, AMC, DS, IP, DW, LJ, JM. All authors read and approve the final manuscript.

## Supplementary Material

Additional file 1 **PRISMA Checklist**. A checklist of items reported in the review. Click here for file

Additional file 2 **Formulae of calculation of outcomes**. A description of the formulae through which outcomes were calculated. Click here for file

Additional file 3 **Description of included studies**. Studies included in the review. Click here for file
