# Supplementary material for: A Meta-analysis of the effects of Exercise Training on Left Ventricular Remodeling Following Myocardial Infarction: Start early and go longer for greatest exercise benefits on remodeling
Source: Trials. 2011 Apr 4;12:92. doi: 10.1186/1745-6215-12-92 (PMC3083361; doi:10.1186/1745-6215-12-92)
Supplement: Additional file 2 — Formulae of calculation of outcomes. A description of the formulae through which outcomes were calculated. [file 1745-6215-12-92-S2.DOC]

**Additional File 2: Formulae for calculation of outcomes**

To calculate outcomes from the studies, an effect size and standard deviation was calculated for pre-post contrast for all outcomes from each trial using the following method{Becker, 1988. #1;Lipsey, 2001 #2}: 1)
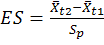

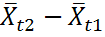
and 2)
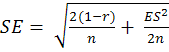
, where
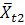
 is the mean at time 2,
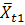
 is the mean at time 1, and
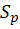
 is the pooled standard deviation of the time 1 and time 2 scores calculated in the standard way (
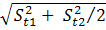
 ),
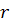
 is the correlation between time 1 and time 2 scores and *n* is the number of subjects on whom measures have been repeated*.* While standard deviations for both time 1 and time 2 scores were available from which to calculate the pooled standard deviation
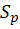
, the correlation between time 1 and time 2 scores
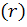
 was not. Therefore, for these analyses, r was assumed, conservatively, to be 0. For each included trial, two such effect sizes were calculated, one for the experimental group and one for the control group. These were combined into a single effect size measure for each reported trial as follows:
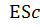

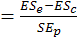
, where
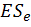
 is the effect size calculated from equation 1 for the experimental group,
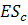
 is that calculated from the control group, and
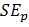
 is the pooled estimate of the standard error estimated in the standard way from the standard errors of the effect sizes of the experimental and control groups calculated using equation 2. In the following the
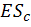
 is referred to as the effect size difference.
