# Supplementary material for: A Meta-analysis of the effects of Exercise Training on Left Ventricular Remodeling Following Myocardial Infarction: Start early and go longer for greatest exercise benefits on remodeling
Source: Trials. 2011 Apr 4;12:92. doi: 10.1186/1745-6215-12-92 (PMC3083361; doi:10.1186/1745-6215-12-92)
Supplement: Additional file 3 — Description of included studies. Studies included in the review. [file 1745-6215-12-92-S3.DOC]

**Additional File 3: Description of included studies**

| Study | Sample  size | Age | Men  % | MI location  % | Time post infarct to exercise, wk | Meds, % | Thrombolysis (%)/angioplasty(%) | Method to assess EF & Pre/Post EF mean (SD) |
| --- | --- | --- | --- | --- | --- | --- | --- | --- |
| Giallauria et al [6] | EX: 30  CN: 30 | 59  58 | 77  80 | ANT-SEP: 53  INF: 33  Other: 14  ANT-SEP: 50  INF: 36  Other: 14 | 0.86 | ACE: 76  ARB: 26  ASA: 96  BB: 53  LL: 76  ACE: 73  ARB: 26  ASA: 93  BB: 53  LL: 73 | 76/100  73/100 | ECHO  Pre: 43.7 (4.2)  Post: 46.2 (4.4)  Pre: 44.7 (4.4)  Post: 42.2 (4.5) |
| Giallauria et al 2008 [5] | EX: 30  CN: 31 | 56  55 | 73  71 | ANT-SEP: 60  INF: 30  Other: 10  ANT-SEP: 55  INF: 29  Other: 16 | 1.0 | ACE: 73  ARB: 23  ASA: 93  BB: 46  LL: 76  ACE: 71  ARB: 26  ASA: 87  BB: 48  LL: 71 | 74/100  74/100 | ECHO  Pre: 41.6 (11.3)  Post: 42.7 (8.3)  Pre: 42.0 (7.6)  Post: 38.2 (8.3) |
| Giallauria et al 2006 [12] | EX: 22  CN: 22 | 55  54 | 86  91 | ANT-SEP: 73  INF: 18  Other: 9  ANT-SEP: 68  INF: 27  Other: 5 | 1.4 | ACE: 95  ANTI-P: 64  ASA: 100  BB: 73  DIU: 82  LL: 100  NIT: 45  ACE: 95  ANTI-P: 59  ASA: 100  BB: 77  DIU: 86  LL: 100  NIT: 55 | 100/90  100/100 | ECHO  Pre: 45.0 (4.0)  Post: 46.6 (3.1)  Pre: 45.0 (3.2)  Post: 45.0 (2.9) |
| Giallauria et al 2006 [13] | EX: 20  CN: 20 | 69  68 | 80  85 | ANT-SEP: 80  ANT-SEP: 75 | 1.6 | ACE: 70  ANTI-P: 20  ARB: 30  ASA: 100  BB: 70  CCB: 30  DIU: 60  LL: 95  NIT: 10  ACE: 65  ANTI-P: 15  ARB: 35  ASA: 100  BB: 75  CCB: 35  DIU: 65  LL: 90  NIT: 15 | 10/90  5/95 | ECHO  Pre: 44.8 (4.0)  Post: 44.6 (1.5)  Pre: 44.8 (2.1)  Post: 44.5 (1.6) |
| Giannuzzi et al [7] | EX: 39  CN: 38 | 54  53 | WG: 95% | WG: 78% ANT | 3.5  3.4 | ACE: 77  ANTI-A: 8  BB: 49  CCB: 8  DIG: 10  DIU: 31  LL: 13  ACE: 79  ANTI-A: 13  BB: 45  CCB: 16  DIG: 10  DIU: 34  LL: 16 | 74/NR  71/NR | ECHO  Pre: 34 (5)  Post: 38 (8)  Pre: 34 (5)  Post: 33 (7) |
| Koizumi et al [14] | EX: 14  CN: 15 | 54  59 | 93  87 | NR  NR | 4.0 | ACE: 36  ANTI-P: 86  ASA: 100  BB: 0.1  CCB: 50  LL: 50  NIT: 100  ACE: 27  ANTI-P: 87  ASA: 100  BB: 0  CCB: 40  LL: 60  NIT: 100 | NR/100  NR/100 | ECHO  Pre: 56 (5)  Post: 57 (5)  Pre: 54 (5)  Post: 54 (5) |
| Kubo et al 2004 [9] | EX: 24  CN: 24 | 59  62 | 88  71 | ANT: 100  ANT: 100 | 4.0 | ACE: 50  BB: 4  CCB: 25  DIU: 38  NIT: 92  ACE: 50  BB: 13  CCB: 38  DIU: 21  NIT: 92 | NR/100  NR/100 | LVG  Pre: 43 (8)  Post: 43 (9)  Pre: 44 (11)  Post: 46 (8) |
| Dubach et al 1997 [11] | EX: 1  CN: 13 | 56  55 | 100  100 | ANT: 50  INF: 33  POST:  ANT: 62  INF: 23  POST: 15 | 5.2  5.0 | ACE: 100  DIG: 67  DIU: 50  ACE: 100  DIG: 54  DIU: 54 | NR/17  NR/8 | MRI  Pre: 38.0 (9)  Post: 38.2 (10)  Pre: 37.0 (10)  Post: 38.3 (13) |
| Giannuzzi et al 1993 [10] | EX: 49  CN: 46 | 51  50 | 100  100 | ANT: 100  ANT: 100 | 5.4  5.2 | ACE: 10  ANTI-A: 4  ANTI-C: 4  ANTI-P: 96  BB: 78  CCB: 10  DIU: 8  NIT: 16  ACE: 28  ANTI-A: 7  ANTI-C: 4  ANTI-P: 96  BB: 70  CCB: 7  DIU: 7  NIT: 26 | 65/NR  76/NR | ECHO  Pre: 51 (14)  Post: 54 (14)  Pre: 48 (13)  Post: 50 (16) |
| Heldal et al 2000 [20] | EX: 19  CN: 18 | 53  52 | 100  100 | ANT: 47  ANT: 61 | 5.4  5.1 | BB: 100  OM: NR  BB: 100  OM: NR | 74/NR  61/NR | RNV  Pre: 44 (9)  Post: 47 (9)  Pre: 45 (8)  Post: 47 (7) |
| Jette et al 1991 [19] | EX, EF <30%: 7  CN, EF <30%: 8  EX, EF 31-50%: 11  CN, EF 31-50%: 10 | 54  51  46  52 | 100  100  100  100 | ANT: 100  ANT: 100  ANT: 100  ANT: 100 | 6.9  6.8  6.4  6.1 | ACE: 29  ANTI-A: 29  BB: 43  CCB: 29  COMBT: 100  DIG: 100  DIU: 86  NIT: 86  ACE: 25  ANTI-A: 38  BB: 38  CCB: 50  COMBT: 100  DIG: 75  DIU: 88  NIT: 100  ACE: 0  ANTI-A: 9  BB: 73  CCB: 18  COMBT: 91  DIG: 45  DIU: 73  NIT: 100  ACE: 10  ANTI-A: 20  BB: 50  CCB: 40  COMBT: 90  DIG: 40  DIU: 40  NIT: 90 | NR/NR  NR/NR  NR/NR  NR/NR | RNV  Pre: 23.9 (3.5)  Post: 28.2 (7.7)  RNV  Pre: 25.3 (4.4)  Post: 32.4 (10.9)  Pre: 39.5 (5.7)  Post: 41.3 (8.9)  RNV  Pre: 40.0 (7.4)  Post: 46.4 (8.4) |
| Grodzinski et al 1987 [21] | EX: 53  CN: 46 | GP: 94 | GM: 48 | ANT: 47  INF: 53  ANT: 48  INF: 52 | Group mean: 6.5 | ANTI-A: 6  ANTI-H: 0  BB: 45  DIG: 32  CCB: 17  COMB: 76  DIU: 0  NIT: 91  ANTI-A: 4  ANTI-H: 2  BB: 67  CCB: 7  COMB: 72  DIG: 46  DIU: 2  NIT: 94 | NR/NR  NR/NR | RNV  Pre: 50.9 (12.8)  Post: 50.2 (12.9)  Pre: 49.0 (10.9)  Post: 52.0 (12.3) |

(ACE: Angiotensin converting enzyme inhibitor; ANT: Anterior; ANT-SEP: Antero-septal; ANTI-H: Anti-hypertensive; ANTI-A, Anti-arrhythmic; ANTI-C: Anti-coagulant; ANTI-P: Anti-platelet; ARB: Angiotensin receptor blocker; ASA: Aspirin; BB: Beta blocker; CCB: Calcium channel blocker; COMBT: Combination therapy; CN: Control; DIG: Digitalis; DIU: Diuretic; ECHO: Echocardiography; EF: Ejection fraction; EX, exercise; GM, group mean; GP, group; INF: Inferior; LL: Lipid lowering agent; LVP: Biplane left ventriculography; MRI: Magnetic resonance imaging; NIT: Nitrates or Nitroglycerin; NR; Not reported; OM: Other medications; Post, posterior; RNV; Radionuclide ventriculography; WG, whole group).
